# Surgery

**Published:** 1895-02

**Authors:** 


### SURGERY.

EDITED BY FLOYD W. McRAE, M.D.

### SPECIFIC POINTS IN ANTISEPTIC SURGERY.

1. Remember to boil instruments for thirty minutes before an operation, when time permits.

2. Remember to use clean, filtered, boiled water in all operations.

3. Remember to immerse instruments only in a carbolic or listerine solution; the mercurial solution blackens the instruments and ruins edges.

4. Remember not to dip soiled cotton in an antiseptic solution, after it has been about the wound; use a new piece.

5. Remember not to touch a sponge or instrument that has fallen upon the floor; it might get back into the vicinity of the wound again.

6. Remember to put the suture or ligature back into the antiseptic solution when it has touched anything that is not aseptic.

7. Remember that the surgeon and his assistants are to scrub, cleanse, and disinfect their hands and forearms before an operation.

8. Remember that the vicinity of the wound is to be shaved, scrubbed, cleansed, and disinfected.

9. Remember not to use a rubber bandage in forcing blood out of a part, like a gangrenous or tuberculous limb, but simply apply a constrictor above the point of disease.

10. Remember to use no antiseptic upon the peritoneum or any of the viscera; if the operation was aseptic, antiseptics could do no good; if the operation was not aseptic, antiseptics could not make it septic.

11. Remember to try always to arrest a bleeding artery by torsion, only using the ligature as a *dernier ressort.*

12. Remember that venous and capillary hemorrhage can be arrested by the application of a hot mercurial lotion to the bleeding surface.

13. Remember to use cocaine in amputating toes, fingers, etc., and in circumcision.

14. Never give ether when albumin is present in the urine, or when there is any pulmonary trouble.

15. Remember always to open an abscess by a free incision, wash out abscess cavity with a carbolic solution (1 to 10), insert a perforated drainage-tube in the deepest part, and apply a moist antiseptic dressing.

16. Remember to wash gun and pistol-shot wounds in a mercurial lotion (1 to 3,000), and pack the wound with iodoform gauze, so that it can heal from the bottom.

17. Remember to use black pins in surgical dressings; they will not rust and can be more readily seen.

18. Remember to let all rubber goods, such as catheters, rubber bandages, fountain syringes, rubber sheets, rubber bags, rubber aprons, dry before they are put away.

19. Remember to boil all instruments that have been used about a person with cancer, syphilis, or tuberculosis in a carbolic solution (1 to 30).

20. Remember always to cleanse a hypodermic needle, and run a piece of wire through it after it has been used.

21. Remember to thoroughly wash, cleanse, and dry all instruments after each operation.

22. Remember to use gauze sponges, which can be thrown away after they have been used.

23. Remember to use silk in suturing parts about the face.

24. It is not necessary to have a case full of instruments to perform an operation, only a few are at times necessary.

25. Remember always to cleanse and disinfect a catheter after it has been used; the most common source of infection of the bladder is by an unclean catheter.

26. Remember to use moist antiseptic dressing in all septic cases, such as abscesses, bone-felons, lymphadenitis, etc., or where primary union is not expected.

27. Remember to move silk sutures on the third or fourth day.

28. Remember that the edges of a wound have to be in exact apposition, and no turning under is to be present if union by first intention is desired.

29. Remember to dust iodoform over the line of sutures covered by iodoform gauze, cotton, tissue, and a rubber bandage.

30. Remember to use the fingers in anointing instruments instead of putting the instruments which are soiled into the ointment.—*Med. Record.*

---

### SUPRAPUBIC PROSTATECTOMY.

Robson (*British Medical Journal*, July 14, 1894) reports twelve cases of operation by this method. He considers this operation in properly selected cases one attended with less danger than is usually thought, and that if thoroughly and completely performed it is capable of affording such relief as may be in many instances genuinely termed a cure, and that in a class of cases which until a few years ago were looked on as incurable. As a method of diagnosis, he strongly recommends bimanual examination. In regard to the selection of cases, whenever a patient has no large amount of residual urine, and can be made comfortable by the passage of a catheter at night or night and morning, and where catheterism is well borne and not difficult or distressing, operative treatment is unnecessary. In complete muscular atony, operation is advisable if the atony has existed only a short time; months duration precludes successful operation. The presence of a large amount of residual urine associated with fair vesical contractility, and not diminishing after regular catheterism, if the patient is in a fair condition and is not sufficiently relieved, is a decided indication for prostatectomy. Cystitis associated with pain and irritation during catheterism is an indication for the operation, as is also the presence of calculi or calculous material. Contraindications are, advanced kidney disease, especially associated with greatly diminished secretion of urea; chronic atony; glycosuria; well-marked degeneration of the blood-vessels associated with general senile debility or other organic disease that would render any major operation unwise. In addition

4

to external antisepsis and washing the bladder out with boric solution, the author advises five to ten grains of boric acid and a little saccharin thrice daily for a few days before the operation, so as to render the urine aseptic if possible. He introduces at most only four to six ounces into the rectal bag, in order not to overdistend the rectum and cause rupture or inflammation. The bladder is filled with boric lotion till it is felt above the pubes. The peritoneum can usually be avoided; but when it must be cut into, it should be dissected up and sutured before the bladder is opened. McGill's scissors or Jessop's cutting ring-forceps are used to remove the portion of prostate desired. Suprapubic drainage has been found sufficient in all cases. In the after-treatment boric acid is given thrice daily, and the bladder is washed out by syringing a solution of boric acid through the urethra to the drainage opening. The drainage-tube is removed on the third day, if possible, and the patient is allowed to sit up within a few days after the operation. Recovery follows without general disturbance.—*American Journal of the Medical Sciences.*

---

### THE BEST TREATMENT OF HEMORRHOIDS.

If the cases seen by the practitioner are sufficiently numerous to justify him in providing himself with the necessary instruments, he will find the clamp and cautery method of treatment an ideal one, and it has not been intended to prefer the ligature to it without some qualification of the statement of preference. While not so simple of performance, it is followed by less distress, and recovery is usually more speedy after it than the ligature. The surgeon who permits his patients to walk out on the fourth day, however, as has been reported, does not decide for their best interest. A week or ten days should elapse, unless an examination shows the wounds healed. If resorted to, two or three precautions are best heeded. Do not use it on tumors high up in the rectum. Open the clamp slowly, and if there is any tendency to bleed, screw up the clamp and again apply the cautery. The cautery is sufficiently hot when dull red, and the part of the stump to which attention should be paid in applying the cautery is that farthest from the operator, that is, where the vessels enter.

Before either operation see that the bowels are thoroughly emptied, and after it introduce an opium suppository.

There are one or two other methods advised, but they are not all that could be desired. One of them, called after the name of an eminent English surgeon, consisting in excision of the "whole of the pile-producing area," deserves to be forgotten, not because it is not simple, but because it is not safe.

A form of hemorrhoidal disease characterized by sessile granulations which bleed easily is best treated by the very old method of applying nitric acid. Introduce a speculum, dry the parts with gauze, and touch the whole granular surface again and again with a bit of cotton moistened with the acid, but containing so little that it will not run over the part not diseased.

Lastly, before beginning any treatment, look out for complications. Especially in women should the pelvic organs other than the rectum be examined. In children, examine the urinary organs.—DR. STEVENS, *Lancet-Clinic.*

---

### POTT'S DISEASE IN CHILDREN.

Pott's disease in children is so often recognized so late in its course that all hope for improvement is futile. The early symptoms and signs which should always be looked for are laid down by Dr. Dillon Brown in the *Archives of Pediatrics* in the following list of different points:

1. The pain, general disability, and sickness are out of proportion to the apparent amount of spinal disease.

2. The onset is alarming and the progress of the disease is more rapid than in tubercular caries—the paralysis being an early symptom and the deformity appearing even in a few weeks after the beginning of the symptoms.

3. The local pain is intense; and the peripheral pains, the deformity, the extreme spinal disability and paralysis, including incontinence of urine and feces, rapidly grow worse in spite of rest in bed and instrumental support.

4. Secondary disease soon appears, rapid emaciation and marked cachexia are seen, and the patient does not live more than six or eight months.

Whether a vertebral caries is due to syphilis or to tuberculosis is of immense importance as regards prognosis and treatment. In both diseases the symptoms are almost identical, and the diagnosis must be based upon the history, the presence or absence of other evidences of syphilis, and the result of treatment. In tuberculosis there is more likely to be an evening rise in temperature, and the pus and débris may contain tubercle bacilli. Syphilis is suggested by nocturnal pains, and the involvement with chronic disease of some other joint or joints or some other part of the spine.—*Cincinnati Lancet.*

---

### ETHER AND ALBUMINURIA.

The *Nouveau Montpellier Medical*, for December 15, contains a review of an article on this subject, by Dr. Barensfeld, which was published in the *Munchener Medicinische Wochenshrift.* The writer remarks that, if etherization has still determined opponents, it is because the careful study of this method of anesthesia has often been somewhat neglected, and because, on account of some real inconveniences of the method, too much stress has been laid on observations of little value.

Certain American surgeons have maintained that ether exercises an unfavorable action on the kidneys, but Barensfeld's researches go to show that this opinion is not very well founded. In order to determine the alleged relation of cause and effect between etherization and albuminuria, Barensfeld analyzed the urine of some persons who had been operated on, before and twenty-four hours after the operation. Out of a hundred and fifty cases albumin was found after the operation in four only, in two of which the patients had shown signs of albuminuria before the operation; moreover, in these two cases the quantity of albumin had not increased under the influence of the ether. With regard to the two cases where albumin did not manifest itself until after the operation, one of the patients had been subjected to laparotomy for intestinal occlusion, after having been for three days in a condition of extreme prostration, and died on the day of the operation. The other one had been operated on for inguinal hernia by Kocher's method, and after the operation considerable albuminuria appeared and persisted, although gradually diminishing, for four days. It was, on the whole, says the writer, the only case out of the one hundred and fifty of true nephritis due to etherization.

The author reports, among others, a very interesting case of nephrectomy for left nephrydrosis in a child of three years, who had borne the ether very well. There had been no vomiting, and after the operation not even a trace of albumin had been found in the urine.—*Jour. Am. Med. Ass'n.*

---

### FIXATION IN THE TREATMENT OF FRACTURES INTO JOINTS.

In an interesting discussion of this subject by Cook (*International Journal of Surgery*, August, 1894) the following conclusions and rules of treatment are laid down:

1. That bony or serious fibrous anchylosis is the result of injury and subsequent inflammation, and not of immobilization.

2. That early passive motion only disarranges the fragments of bone, thereby increasing the production of callus; that it irritates the injured ligaments and, by increasing the inflammation, tends to produce the anchylosis it is thought to prevent.

3. Immobilization is useful only when active inflammation is present, or until the ruptured ligaments and broken bones have thoroughly united.

4. The logical treatment of a fracture into a joint, therefore, should be rest and local applications to reduce inflammation. Reduction of the fracture as early as possible, then immobilization until the bones and ligaments have united (from three to eight weeks, or more, according to circumstances).

5. Passive motion, massage, and use till the tissues become normal, or, if the massage fails, complete rupture of all adhesions under an anesthetic. The factors which will ultimately determine anchylosis are the nature of the original injury, the character and duration of the subsequent inflammation, the destruction of bone and cartilage, cicatricial contraction of the soft tissues around the joint, and the age and condition of the patient.—*Therapeutic Gazette.*

---

### STONE IN THE BLADDER: CHOICE OF OPERATION.

William H. Hingston, M.D., of Montreal, in *Medical News*, says in regard to the choice of operation for stone in the bladder: "Lithotrize in all cases of adults in whom the stone is neither too large nor too hard for the lithotrite; when the urethra is or can be made sufficiently capacious for the crushing instrument; in children, however young, when the urethra permits the introduction of a crushing instrument. In very young children the cutting operation is preferable. The precise age at which lithotrity is possible must vary with the calibre of the canal, which in yonng children varies greatly in its capaciousness and its capacity. When the urethra in the child is not and cannot be made fit to receive the lithotrite, the cutting operation to be chosen is the lateral method. In cases of stone in the aged, when enlarged prostate not only prevents the stone being seized, but its dimensions being ascertained, one should act as if the calculus were of large size and incapable of reduction, and proceed to operate by the suprapubic method."

Surgical interference in cases of calculus in the female remains the same. The method employed years ago by Erichsen, Thompson, and others has since been followed, and stones of large size are removed generally *per vias naturales*, after dilatation.

In exceptionally large calculi the lithotrite commonly suffices; and rarely, indeed, is the surgeon obliged to resort to the knife in the case of females.—*Canadian Practitioner.*

---

### ANESTHESIA FATALITIES IN GERMANY.

For four years, during which careful records have been kept and collected, there have been 166,812 chloroform cases, with 63 deaths (1 in 2,647); 26,320 ether cases, with 2 deaths (1 in 13,160); 8,014 chloroform and ether cases, with 1 death (1 in 8,014); 4,190 A. C. E. mixture cases, with 1 death (1 in 4,190); 7,541 bromide of ethyl cases, with 2 deaths (1 in 3,770); 597 pental cases, with 3 deaths (1 in 199). Figures also seem to show that chloroform is gradually giving way to ether in Germany.

